# The Marriage Relation

**Published:** 1857-07

**Authors:** 


### THE MARRIAGE RELATION.

THAT the relation of marriage is the more natural condition of man, and, in the main, promotes happiness and long life, is demonstrated in the double fact, that unmarried adults do not live as long as an equal number of married people; and that there are more, insane single persons in our asylums, in proportion, than of married.

Whatever then promotes marriage, and tends to make that relation permanent, does to that extent advance the well being of the race. Hence the Scriptures hedge up the way of escaping from that relationship, when once formed, and admits of but one ground for divorce. This is one of the plainest intimations, that the causes of unhappiness between man and wife, when such exist, should be removed, not by dissevering the relation, but by the large cultivation of those virtues, the germs of which are found in every human bosom: to wit, a patient forbearance, and an affectionate sympathy as to each other's faults, errors, and weaknesses.

We commend these views to the consideration of those of our exchanges which are devoted to the revolution of our social condition, with a view to promote "*Human Progress and Well-being*," and whose "Tracts for Thinkers," as to the philosophy of Woman's Rights, Dress, Reform, Free Love, and kindred topics, are thrown broadcast over the community. We do not doubt their motives. We are willing to believe that they are sincere in their efforts for human advancement. But we are satisfied that the same intelligence, tact, and energy directed to the ordinary channels of undisputed philanthropy, would tell far more efficiently for the happiness of mankind. We do not think that we would promote public morals by a more definite requested "notice" as to the place, name, and terms of their publications.

